# *In Vitro* Regeneration of Decellularized Pig Esophagus Using Human Amniotic Stem Cells

**DOI:** 10.1089/biores.2019.0054

**Published:** 2020-02-21

**Authors:** Nikhil B. Nayakawde, Ketaki Methe, Debashish Banerjee, Malin Berg, Goditha U. Premaratne, Michael Olausson

**Affiliations:** ^1^ Laboratory for Transplantation and Regenerative Medicine, Sahlgrenska Academy at Gothenburg University and the Sahlgrenska Transplant Institute at Sahlgrenska University Hospital, Gothenburg, Sweden.; ^2^ Department of Otolaryngology, Head and Neck Surgery, and Sahlgrenska Academy at Gothenburg University and the Sahlgrenska Transplant Institute at Sahlgrenska University Hospital, Gothenburg, Sweden.; ^3^ Department of Transplantation Surgery, Sahlgrenska Academy at Gothenburg University and the Sahlgrenska Transplant Institute at Sahlgrenska University Hospital, Gothenburg, Sweden.

**Keywords:** cell adhesion, cell differentiation, de and recellularization, esophagus, stem cells, tissue engineering

## Abstract

Decellularization of esophagus was studied using three different protocols. The sodium deoxycholate/DNase-I (SDC/DNase-I) method was the most successful as evidenced by histology and DNA quantification of the acellular scaffolds. Acellular scaffolds were further analyzed and compared with native tissue by histology, quantitative analysis of DNA, and extracellular matrix (ECM) proteins. Histologically, the SDC/DNase-I protocol effectively produced scaffold with preserved structural architecture similar to native tissue architecture devoid of any cell nucleus. ECM proteins, such as collagen, elastin, and glycosaminoglycans were present even after detergent-enzymatic decellularization. Immunohistochemical analysis of acellular scaffold showed weak expression of Gal 1, 3 Gal epitope compared with native tissue. For performing recellularization, human amnion-derived mesenchymal stem cells (MSCs) and epithelial cells were seeded onto acellular esophagus in a perfusion–rotation bioreactor. In recellularized esophagus, immunohistochemistry showed infiltration of MSCs from adventitia into the muscularis externa and differentiation of MSCs into the smooth muscle actin and few endothelial cells (CD31). Our study demonstrates successful preparation and characterization of a decellularized esophagus with reduced load of Gal 1, 3 Gal epitope with preserved architecture and ECM proteins similar to native tissue. Upon subsequent recellularization, xenogeneic acellular esophagus also supported stem cell growth and partial differentiation of stem cells. Hence, the current study offers the hope for preparing a tissue-engineered esophagus *in vitro* which can be transplanted further into pigs for further *in vivo* evaluation.

## Introduction

Among clinical conditions that demand esophageal replacement surgery, esophageal carcinoma is of particular importance: it is the sixth leading cause of cancer-related mortality and affects almost 500,000 patients every year globally.^1^ Other conditions that need such treatment are congenital esophageal diseases in pediatric patients (esophageal atresia), traumatic conditions, and various end-stage benign esophageal diseases. Current clinical practice for a management of long-gap esophageal defect is with stomach, colon, and small intestine autograft with vascular anastomoses. Such procedure, however, is usually followed by serious postoperative complications, and long-term graft failure problems.

In recent years, tissue-engineered (TE) organs have been suggested as a promising source for conventional transplantation. It has been shown in both preclinical models and in clinical studies that the concept may be ideal to address the issues of donor scarcity and graft rejection. Furthermore, several attempts to construct grafts for patients with esophagus cancer using natural acellular or synthetic scaffolds have been carried out but have often been subject to major surgery with significant serious complications. Organs derived from biological extracellular or synthetic matrices and recellularized with autologous stem cells would fulfill the requirement for immunosuppressant-free transplantation in both whole organ transplantation and in tissue repair. Although TE three-dimensional complex whole organs, such as heart, kidney, lung, and liver, have been successfully used in preclinical studies, their introduction into clinical practice is still awaited.^2,3^ However, decellularized hollow organs, subsequently seeded with autologous stem cells have been successfully used in the clinic. These have included trachea followed up for a period as long as 8 years^4^ and portal vein reported for up to 6 years follow-up.^5^

TE acellular esophageal constructs have been successfully created with de/recellularization technique in experimental studies.^6–8^ Recently, the de/recellularization of the porcine esophagus has been carried out using human and porcine adipose-derived stem cells, however it lacks the complete cellularization of the matrix.^9^ So far, however, the *in vitro* characterization of TE scaffold with muscular and mucosal regeneration is largely lacking.

In the present study, we tested decellularization of porcine esophagus using three different protocols followed by recellularization with human amnion mesenchymal and epithelial cells.

## Materials and Methods

### Preparation of decellularized scaffold

Pig esophagus tissues were taken from deceased (1–4 h after death) healthy Swedish domestic pigs (*n* = 25, weight 45–50 kg) procured from the slaughterhouse. After incision of skin and cervical anterior muscle, the larynx, trachea, common carotid artery, and peripheral tissue were exposed. Esophagus was explanted from the cervical position to the gastroesophageal position, was placed in a sterile phosphate-buffered saline (PBS) solution containing 1% antibiotics and antifungal (penicillin, streptomycin, and amphotericin-B, antibiotic–antifungal, Gibco; Life Technologies, Carlsbad, CA), and stored at −80°C until use. Before use, the esophagus was thawed at 37°C in a water bath and washed thoroughly with PBS containing 1% antibiotics and antifungal. Esophagus was then cannulated and perfused with distilled water (DW) at 3 mL/min for 24 h using a peristaltic pump (ISM-941; Ismatec, Wertheim, Germany). Three different protocols were used to decellularize the porcine esophagus as described below.

### Decellularization protocol 1

Porcine esophagus were decellularized by minor modification of the previously described detergent-enzymatic decellularized protocols.^6,8^ Briefly, the esophagi (*n* = 15) were subjected to a decellularization cycle by perfusion with 4% sodium deoxycholate (SDC; Sigma-Aldrich, St. Louis, MO) at room temperature (RT) for 4 h, followed by 2000 U DNase-I (Roche, Mannheim, Germany) in PBS containing Ca^+^ and Mg^+^ at 37°C for 3 h, and lastly with DW at 3.0 mL/min for 24 h. A PBS washing step (1 h perfusion) was used between the SDC and DNase-I steps to wash away excess SDC remnants from the tissue. The complete perfusion procedure of the esophageal lumen with SDC, DNase-I, and DW solution was repeated three times to completely decellularize the esophageal tissue.

### Decellularization protocol 2

The esophagi (*n* = 5) were subjected to a decellularization cycle by perfusion with 6% Tri-n-Butyl phosphate (TnBP; Sigma-Aldrich) at RT for 4 h, followed by 6% Triton X-100 (Sigma-Aldrich) for 3 h and then with 2000 U DNase-I in PBS containing Ca^+^ and Mg^+^ at 37°C for 3 h, and finally with DW at 3.0 mL/min for 24 h. A PBS washing step (1 h perfusion) was used between the Triton X-100 and DNase-I steps to wash away excess Triton X remnants from the tissue. The whole procedure was repeated for 11 cycles to ensure thorough decellularization of the esophagus.

### Decellularization protocol 3

This protocol is based on nondetergent decellularization. Before ultrasonication (Sonifier 250; Branson, Danbury, CT), the tip and probe were sterilized. The esophagi (*n* = 5) were transferred to an ice-cold PBS and the tip of the probe was passed through the lumen, which was subjected to ultrasonication at a controlled output setting of six for constant duty cycles, each lasting 1 min. These ultrasonication cycles were repeated three times. To complete the decellularization cycle, esophagi were connected to the perfusion pump and perfused with 2000 U DNase-I in PBS containing Ca^+^ and Mg^+^ at 37°C for 3 h, and finally with DW at 3.0 mL/min for 24 h. The total decellularization process consisted of 11 cycles.

All the steps described above were performed under a laminar air flow, except the ultrasonication step. All instruments, plastic wares, and silicon tubing were sterilized by autoclaving before decellularization. At the end of each decellularization cycle, the tissue was biopsied for histology, DNA quantification, and protein quantification. Decellularized esophagi were stored at −80°C until the start of the recellularization procedure.

### Assessment of decellularization

A ring of muscle with mucus lining from decellularized, recellularized, and normal esophageal tissues was fixed in 10% phosphate-buffered formalin after resection for 24 h at RT. Rings were washed in DW, dehydrated in graded alcohol, embedded in paraffin, and sectioned at 4.5–5 μm thickness using a rotary microtome (HM-355S; Microm, Walldorf, Germany). Adjacent sections were saved for further analysis of immunohistochemistry and immunofluorescence staining. Some section slides were stained with Hematoxylin and Eosin (H&E) and 4’,6-diamidino-2-phenylindole (DAPI) to check for the presence of nuclei and morphological changes.

Please refer to Supplementary Data for quantification of DNA and detergent in normal and decellularized esophagus.

### Biochemical assessment of extracellular matrix components

#### Collagen

The amount of soluble and insoluble collagen in the lyophilized tissue sample of normal and decellularized esophagi (*n* = 7) tissues were measured by the Sircol Assay Kit (Biocolor, Carrickfergus, UK). Collagen was extracted by acid–pepsin extraction procedure according to the instruction given with the kit and precipitated by adding acid neutralizing reagent, followed by isolation and concentration reagent. Furthermore, the precipitate was incubated with collagen dye reagent. This complex was further recovered by the addition of alkali reagent and measured at 555 nm on a plate reader. All reagents used were provided with the kit.

#### Glycosaminoglycan

The amount of glycosaminoglycans (GAGs) in the lyophilized tissue sample of normal and decellularized esophagi (*n* = 7) were measured using the Blyscan Sulfated Glycosaminoglycan Kit (Biocolor). The tissue samples were digested with papain extraction reagent provided with the kit at 65°C. The extracted GAGs were then incubated with Blyscan dye reagent to form a precipitate of sulfated GAG dye complex. This precipitate was further dissociated by the dissociation reagent provided with the kit and absorbance was measured at 656 nm on a plate reader.

#### Elastin

The amount of elastin was measured with the Fastin Elastin Assay Kit (Biocolor). The elastin was extracted from the lyophilized tissue sample of normal and decellularized esophagi (*n* = 7) by digestion with oxalic acid at 100°C, and precipitated by using elastin-precipitating reagent provided with the kit. The formed precipitate was incubated with elastin dye reagent, followed by its dissociation in dye dissociation reagent, both provided with the kit. Absorbance was measured at 513 nm on a plate reader.

### Preparation of recellularized scaffold

#### Isolation and expansion of cells for recellularization

Human amnion was collected from discarded human placenta at Sahlgrenska University Hospital/Östra, Gothenburg. A permit was not required from the Regional Ethics Permit Board. Human epithelial and mesenchymal stem cells (MSCs) were isolated from human amniotic membranes according to previously published methods with some modification of the procedure.^10,11^ Detailed information on isolation of epithelial and MSCs is provided in the Supplementary Data.

#### Recellularization in a perfusion–rotation bioreactor

The whole procedure of assembling bioreactor, disinfection, or sterilization of the acellular esophagus, and the seeding of stem cells were carried out aseptically in a laminar air flow. A Harvard prototype bioreactor (Harvard Apparatus, Holliston, MA) was used for the recellularization with slight modification to the perfusion assembly. The bioreactor had a polymeric main chamber, scaffold holder, epithelial media reservoir bottle, driving unit for scaffold rotation, silicon tubing, and a peristaltic pump (Fig. 5C, F). The bioreactor assembly was autoclaved before use. Decellularized esophageal tissues (5–7 cm in length) were disinfected by perfusion with 0.18% peracetic acid (Sigma-Aldrich) in a sterile PBS at 3.0 mL/min (pH 7.2) for 3 h. Esophageal tissues were then washed thoroughly with a sterile PBS for 12 h by gentle agitation on the mechanical shaker. Just before attaching to the bioreactor assembly, esophagus tissues were again soaked in the 70% ethanol for 5 min and washed several times in a sterile PBS. Acellular esophageal tissues (*n* = 5) were tied onto the scaffold holder from both sides using 2–0 sutures. The scaffold holder with attached esophagus was placed in a main chamber of the bioreactor. One end of the silicon tubing was allowed to pass the epithelial medium through the lumen of the esophagus while the other end was connected to the epithelial media reservoir bottle. The esophagus was then perfused with epithelial media at 1 mL/min through the lumen using a peristaltic pump at 0.5 rpm for preconditioning before recellularization. The bioreactor assembly was kept in the incubator (37°C, 5% carbon dioxide [CO_2_]) for 24 h. In total, 300 mL (200 mL external to the lumen, 100 mL internally) culture medium was required for each day.

The epithelial cells at passage 3 were detached from culture flasks by trypsinization, and the cell pellet diluted with 2 mL Dulbecco's modified Eagle's medium (DMEM; 70 × 10^6^ cells per mL), to inject into the lumen of the esophagus through a three-way cannula. Subsequently, MSCs in passage 5 were detached from the culture flask by trypsin treatment, diluted with 2 mL DMEM (70 × 10^6^ cells per mL) and applied longitudinally to the external surface of the matrix with a syringe. Cells were allowed to attach to the scaffold for 1 h at 0.5 rpm (scaffold rotation) in the laminar air flow bench. After a 1-hr incubation period, the bioreactor was moved aseptically into the incubator (37°C, 5% CO_2_). The cell culture medium was added (200 mL externally, 100 mL internally) and the rotation was started at 0.5 rpm. The following day, the perfusion culture was resumed and the flow rate was gradually increased from 1 to 3 mL/min during the recellularization process. The flow was maintained at 3 mL/min for 2 weeks in the bioreactor while keeping the rotation constant throughout the process (0.5 rpm). The external medium (mesenchymal growth) was changed every 24 h and the internal medium (epithelial cells) every 12 h. A sample of recellularized tissue was taken aseptically at days 4, 7, and 14 before being processed for histological analysis.

#### Quantification of seeded cell, cell proliferation, and apoptotic marker

To quantify apoptotic cells in the recellularized tissue samples, tissue samples (*n* = 5) were taken at days 4, 7, and 14 during the recellularization procedure and processed them for histological evaluation. The Click-iT Plus 488 (Life Technologies Corp, Eugene, OR) *in situ* apoptosis detection kit was used for terminal deoxynucleotidyl transferase dUTP nick end labeling (TUNEL) assay according to the manufacturer's instruction. The samples were treated with Alexa 488, the dye supplied with the kit to visualize fragmented DNA in green color. Samples were then washed twice in PBS, and counterstained with DAPI with mounting media. Normal pig esophageal samples were used as positive control samples by treating the samples with 1 U of DNase-I to induce DNA strand breaks.

Proliferation marker, Ki67 expression in the recellularized esophagus was analyzed by immunofluorescence staining as mentioned in the histology and immunohistochemistry section. Sections were probed with secondary antibody Alexa 594 dye and counterstained with DAPI containing mounting media.

The number of apoptotic cells, proliferative cells, and total nuclei was calculated by taking micrographs of 10 randomly selected fields in the five recellularized grafts at days 4, 7, and 14. Micrographs were taken at 200 × magnification on an advanced Leica fluorescence microscope. CellProfiler software (version 2.2.0) was used to calculate the total number of cells and positive cells as primary object and the secondary object, respectively.

#### Immunohistochemical detection of de- and recellularized scaffold

Structural proteins, functional proteins, and GAGs expression in decellularized tissues were evaluated by immunohistochemistry, immunofluorescence, Masson's trichrome (MT; ScyTek Laboratories, Inc., West Logan, UT), and Modified Russell-Movat's pentachrome (MP) staining (ScyTek Laboratories, Inc.). Immunohistochemistry was performed by the ImmPRESS Peroxidase-Based Polymer Detection Kit (Vector Laboratories, Burlingame, CA) for anti-mouse and anti-rabbit immunoglobulin G. Briefly, antigen retrieval was achieved by incubating the slides in 10 mM citrate buffer, pH 6.0 in a thermostatic bath at 95°C for 30 min. Tissue sections were treated with 3% hydrogen peroxide in DW to quench the endogenous peroxidase activity followed by blocking with 2.5% normal horse serum (supplied with the kit), primary antibody (Supplementary Table S1 for antibody list) diluted in 2.5% normal horse serum, ImmPRESS regent, and lastly the Vector Brown Chromogen Kit (Vector Laboratories) according to the manufacturer's instructions.

Immunofluorescence staining was performed by incubating tissue sections in antigen retrieval buffer (10 mM citrate buffer, pH 6.0) at 95°C for 30 min. Tissue sections were then blocked with 5% blocking serum (goat serum) in 1% bovine serum albumin (BSA) before adding primary antibody. Slides were then incubated in the primary antibodies (Supplementary Table S1 for antibody list) diluted in 1% BSA and stored overnight in the fridge. After washing three times with PBS-Tween, slides were then incubated for 50 min at RT in the secondary antibody (Supplementary Table S1 for antibody list and dilutions). Finally, slides were washed three times with PBS-tween in the dark and mounted with nuclear counterstain with DAPI (Abcam, Cambridge, UK). For three color staining, a cocktail of two primary antibodies (mouse anti-pig/human and rabbit anti-pig/human) was used. Two secondary antibodies (dilution 1:300 to 1:500) with different excitation wavelengths (Alexa 488 and 594) were simultaneously incubated. Negative controls were processed by replacing the primary antibody with diluents only on each slide. Normal pig esophageal tissues and appropriate positive control tissues were also used according to primary antibody data-sheets to detect possible nonspecific signals in the staining. MT and MP staining were performed to detect collagen, elastic fibers, and mucin in the tissue according to the manufacturer's instruction. Micrographs were taken on a Leica microscope DM5500B with AF 6000 LX software (Germany) installed.

### Statistical analysis

Results are presented as mean ± standard error of mean. When normal distribution could not be demonstrated, the Mann–Whitney *U*-test was used to compare groups. Kruskal–Wallis test was used when comparing three or more groups, followed by Dunn's multiple comparison *post hoc* test to check statistical difference between the groups. We also used the D'Agostino-Pearson omnibus test to check for normal distribution and the Shapiro–Wilk test for sample sizes <8. The statistical analysis was carried out by GraphPad Prism 8.2 software. The statistical analysis in each experiment is defined in the corresponding figure legend.

## Results

### Esophagus decellularization and characterization

**FIG. 1.**
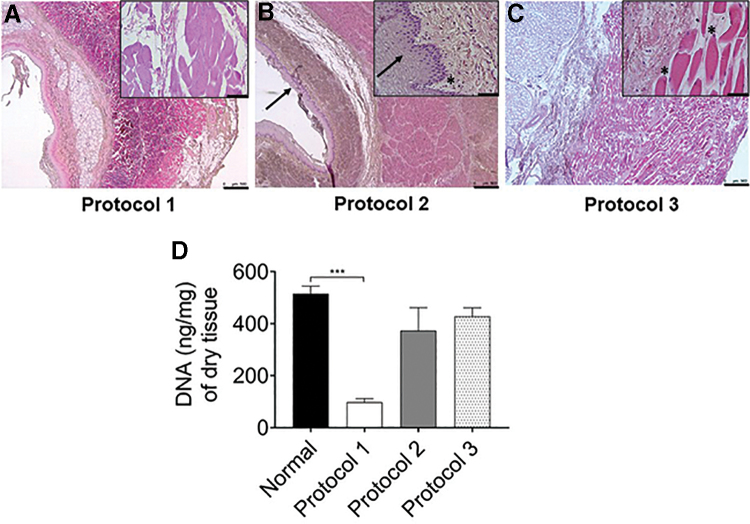
Comparison of different decellularization protocols. **(A)** H&E staining of the decellularized esophageal graft. **(A)** Protocol 1 shows preservation of tissue structures but absence of blue cell nuclei. However, incomplete decellularization of tissues sections of protocol 2 **(B)**, and protocol 3 **(C)** showing prominent blue-colored cell nuclei (asterisk) and an intact squamous epithelial cell layer (arrow) in lumen. DNA quantification of the normal and decellularized esophagus **(D)** showed 82% of DNA is removed in protocol 1 (*n* = 7), but no significant difference was found in protocols 2 and 3 compared with normal tissue. **A** (*n* = 9), **B** and **C** (*n* = 5). Scale bars: 500 μm **(A–C)**, 50 μm (insets with higher magnification). Error bars represents SEM; normal and protocol 1 (*n* = 7), protocols 2 and 3 (*n* = 5). Kruskal–Wallis test followed by Dunn's multiple comparison *post hoc* test was used to calculate *p*-value. ***Represents *p* = 0.0008. H&E, hematoxylin and eosin; SEM, standard error of mean.

We compared three different protocols for decellularization of the porcine esophagus. H&E staining and DNA quantification tests on the tissue pieces obtained from the three protocols were performed. Protocol 1 (SDC/DNase-I) was efficient in terms of removing nuclear material from the tissue, whereas protocols 2 and 3 were unable to remove cell nuclei from the tissue even after 11 decellularization cycles (Fig. 1A). In sections from protocols 2 and 3, clear signs of intact cells were observed in the muscular network, epithelial lining, and mucosal area of the esophagus in H&E-stained tissue (Fig. 1B, C). Quantification of double-stranded DNA in protocol 1 decellularized scaffold was significantly less (*p* = 0.0008) than that of native esophagus (Fig. 1D), whereas, protocol 2 had 72% and protocol 3 83% of donor DNA left at the end of decellularization cycles (Fig. 1D). Therefore, protocol 1-derived decellularized esophagus was used for further extracellular matrix (ECM) protein analysis and recellularization.

**FIG. 2.**
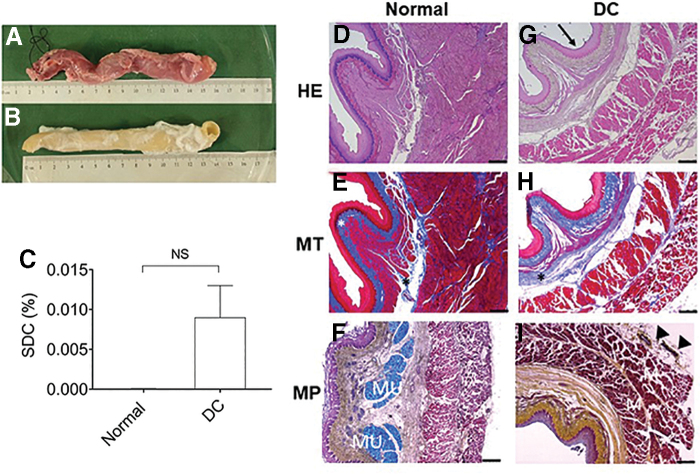
Macroscopic and microscopic analysis of protocol 1 decellularized esophagus; and detergent quantification of the esophagus. Native esophagus **(A)** and decellularized esophagus appeared whitish but similar in size and shape to the native esophagus **(B)**. The esophagus was perfused through the lumen as mentioned in protocol 1. **(C)** Normal and decellularized esophagus tissue samples were lysed and 100 μg of protein from lysates were used for measurement of SDC concentration following reaction with concentrated sulfuric acid at 389 nm. Microscopically, decellularized esophagus **(G–I)** shows preservation of the tissue architecture, with prominent epithelial network without cell nucleus (arrow) compared with the normal esophagus **(D–F)**. MT stain shows the presence of blue collagen (asterisk) in the decellularized tissue compared with the native tissue **(E, H)**. MP staining showing the presence of black elastic fibers (arrowhead), red muscle (M) tissue, but absence of blue-colored mucin (MU) in decellularized tissue compared with the normal tissue **(F, I)**. (*n* = 9); Scale bars: 500 μm **(C–H)**. Error bars represents SEM; normal and decellularized (protocol 1; *n* = 5). Mann–Whitney test was used to calculate *p*-value. DC, decellularized; MP, Movat's pentachrome; MT, Masson's trichrome; NS, not significant; SDC, sodium deoxycholate.

At the end of decellularization, acellular esophageal scaffold appeared white and patent retaining the overall shape and macroscopic structure of the native esophagus (Fig. 2A, B). Histological examination showed complete removal of nuclear staining but preservation of the pink eosinophilic staining typical of collagen in acellular scaffold, as compared with the normal esophagus (Fig. 2D, G). The scaffold architecture of esophagus, particularly the vessels, mucosal network, muscularis mucosa, muscularis externa, and adventitia were well preserved after the decellularization, confirmed by H&E, MT, and MP staining (Fig. 2D–I). MT stain showed the presence of blue-colored collagen fibers in the basement membrane, lamina propria, submucosa, around the blood vessels, muscularis externa, and adventitia as seen in the native esophagus (Fig. 2E, H). However, MP-stained blue-colored mucus glands appeared in submucosa of the native esophagus but not in the decellularized tissue, which suggests effective decellularization in the center of the esophagus tissue (Fig. 2F, I). MP also stained elastic fibers (black) in lamina propria, between muscularis mucosa and muscularis externa, and around the blood vessels.

The SDC quantification did not show any significant difference between normal and decellularized tissue. In the decellularized tissue, a residual detergent concentration was negligible (0.009% ± 0.004; Fig. 2C). The negligible SDC concentration in the tissue suggested that even after 12 h of total exposure to SDC, detergent did not accumulate in the acellular tissue of esophagi.

**FIG. 3.**
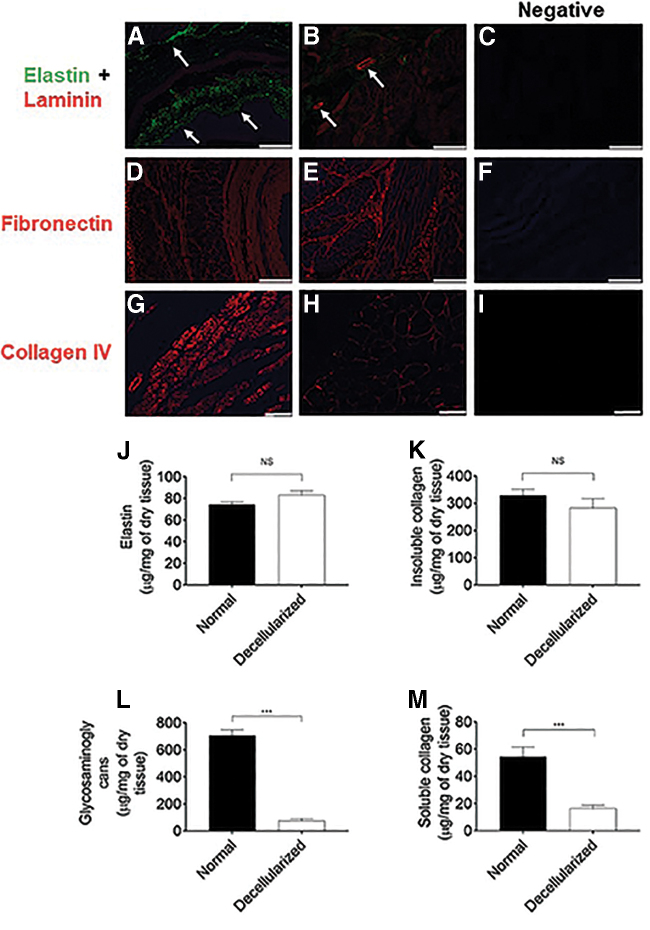
Characterization of decellularized esophagus by immunofluorescence staining and ECM protein quantification. **(A, B)** Green-colored elastin (white arrows) is well retained after the decellularized process but laminin (red) expression is weaker in the tissue and mostly present around the blood vessel structure **(**white arrows in **B)**. However, fibronectin **(D, E)** and collagen **(G, H)** show intense red staining in the tissue. **(C, F, I)** Negative control shows no fluorescent staining in the tissue. DAPI counterstain (blue) is negative in all tissue sections, which is another indication of nucleus-free esophageal tissue. (*n* = 9). **(J–M)** Normal and decellularized porcine esophageal tissue samples were also subjected to quantification of the extracellular matrix protein, elastin by Fastin assay, glycosaminoglycans by Blyscan assay, and insoluble and soluble collagen by the Sircol Assay Kit (*n* = 7). The results represent mean ± SEM, where *p* < 0.05 was considered as statistically significant using Mann–Whitney test; NS, ***Represents *p* = 0.0006. Scale bars: 500 μm **(A–D, F)**, 250 μm **(E)**, and 100 μm **(G–I)**. DAPI, 4’,6-diamidino-2-phenylindole; ECM, extracellular matrix.

Elastin, collagen IV, and fibronectin were well preserved with an almost complete network of these proteins after the decellularization process (Fig. 3A–I), as determined by immunofluorescence staining. Higher expression of elastin in the decellularized tissue further confirms predominant preservation of elastic fibers in the tissue (Fig. 3A). However, laminin only appeared in a patchy fashion, mostly near probable footprints of smooth muscle fibers and around the blood vessels in the tissue (Fig. 3B). Expression of collagen types II, III, and V was absent both in normal and the decellularized scaffold. By using dry tissues of normal and decellularized esophagus, the amount of elastin, GAGs, insoluble collagen, and soluble collagen were estimated by biochemical assay. Elastin in the decellularized tissue was found to be well preserved compared with normal tissue (*p* = 0.07; Fig. 3J). These findings were consistent with the immunofluorescence analysis for elastin. In contrast, GAGs in the decellularized tissue were depleted by 90%, as compared with that in the normal tissues (*p* = 0.0006; Fig. 3L). Insoluble collagen was well preserved in the decellularized tissue compared with the normal tissue (283.3 ± 34 μg/mg in decellularized vs. 329 ± 22 μg/mg in normal, not significant; Fig. 3K). Compared with normal tissue, soluble collagen in the decellularized tissue was reduced by 70% (*p* = 0.0006; Fig. 3M).

**FIG. 4.**
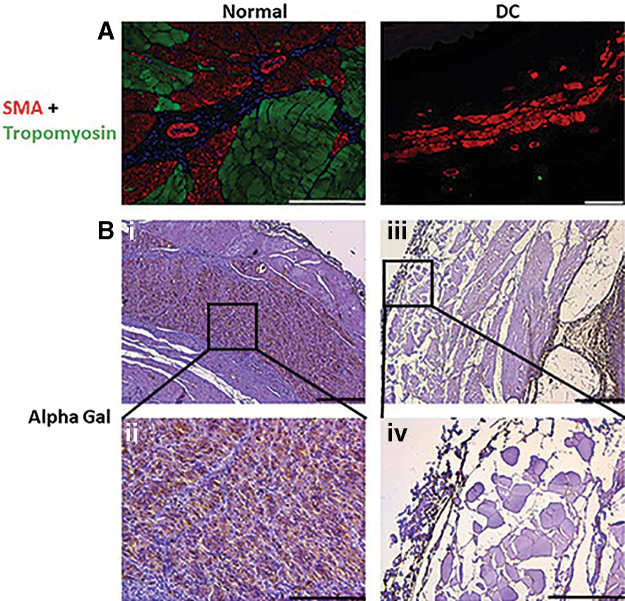
Expression of cellular proteins in the normal and decellularized esophagus by immunofluorescence. **(A)** Expression of SMA in red and Tropomyosin (green) in the normal tissue and decellularized tissue. SMA (red) is still present in decellularized esophagus. However, tropomyosin (green) is not retained in the tissue. Slides were counterstained with DAPI for blue-colored nuclear stain. **(B)** Strong expression of alpha gal (brown) in the normal esophagus tissue in lower **(i)** and higher magnification **(ii)**. Whereas, alpha gal expression is weaker in the decellularized esophagus lower **(iii)** and higher magnification **(iv)**. (*n* = 5). Scale bars: 200 μm **(A)**, 500 μm **B (i, iii)**, and 200 μm **B (ii, iv)**. SMA, smooth muscle actin.

Interestingly, we found a strong expression of smooth muscle actin (SMA) and weak expression of tropomyosin in the decellularized esophagus when compared with the native tissue (Fig. 4A). Immunohistochemistry analysis revealed the presence of Gal epitopes on the normal esophageal tissues, whereas expression of the Gal epitope was weak in the decellularized tissue (Fig. 4B-i to B-iv).

### Amnion-derived stem cell isolation, expansion, and characterization

**FIG. 5.**
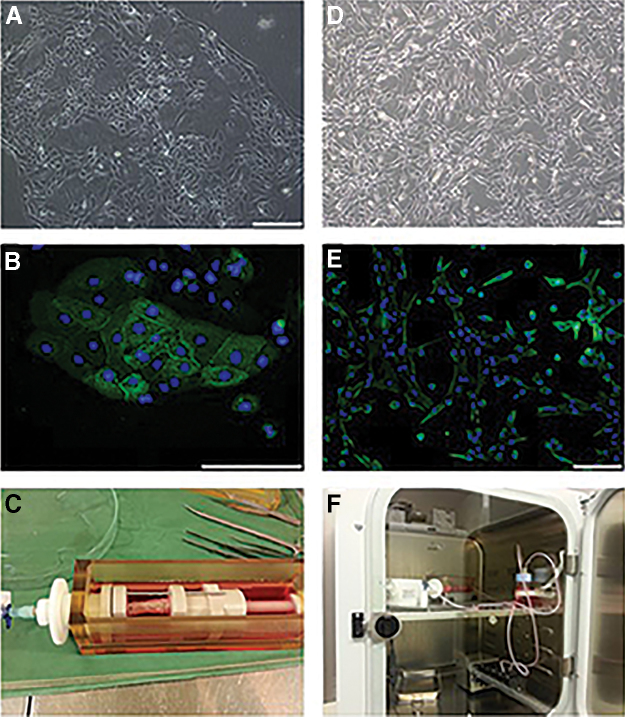
Stem cell expansion, characterization, and recellularization setup. **(A)** Human amnion-derived epithelial cells in culture and **(B)** strong expression of epithelial cell adhesion molecule (green), epithelial cell marker in these cells at passage 3. **(D)** Cultured human amnion-derived MSCs and its positive staining for MSC marker vimentin (green; **E)** in immunofluorescence microscope at passage 5. Nuclei (blue) were counterstained with DAPI. **(C)** A perfusion–rotation Harvard prototype bioreactor showing organ-holding chamber with recellularized esophagus **(F)** and media reservoir connected to the main chamber connected through a silicone tubing. Scale bars: 200 μm **(A, B, D, E)**. MSCs, mesenchymal stem cells.

Human amnion-derived epithelial and mesenchymal cells were isolated and grown in culture plates with specialized media until sufficient numbers were obtained for recellularization. In culture, epithelial cells had a typical cuboidal shape and upon staining, most of the cells showed expression of the epithelial marker epithelial cell adhesion molecule (Fig. 5A, B). However, the expression of the epithelial marker declined after passage 3 in culture, and more vimentin-positive cells took over in the culture plates (data not shown). Moreover, MSCs showed positive staining for vimentin before seeding onto the scaffold (Fig. 5D, E).

### Bioreactor set-up, cell seeding, recellularization, and characterization of engineered esophagus

**FIG. 6.**
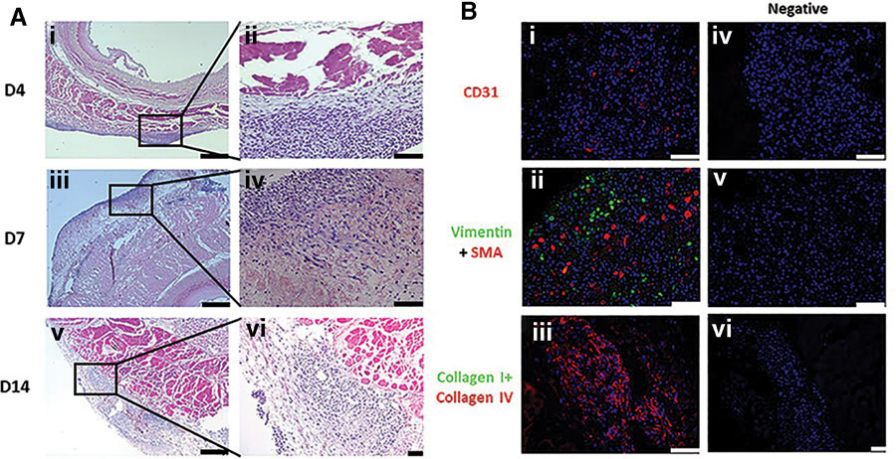
Microscopic characterization of the recellularized esophagus. **(A)** H&E staining showing growth of mesenchymal cells (blue nuclei) in the esophageal tissue at days 4, 7, and 14 at low **(i, iii, v)** and high **(ii, iv, vi)** magnification. **(B)** Very few seeded stem cells are differentiated into endothelial cells **(i)** however, vimentin and SMA expression was abundant in the recellularized esophageal tissue **(ii)**. Collagen I was absent but collagen IV expressed in the recellularized esophagus **(iii)**. Negative control shows no fluorescence stain for markers but nuclear stain DAPI is positive in recellularized tissues **(iv–vi)**. Scale bars: 750 μm **A (i, iii, v)**, 100 μm **A (ii, iv, vi)** and **B (i–vi)**.

Both amnion-derived epithelial and MSCs were seeded onto the acellular esophagus connected to the bioreactor scaffold holder (Fig. 5C, F). Vasculature of the decellularized esophagus consisted of very small arteries and veins. Since it was difficult to inject stem cells through these tiny blood vessels of the esophagus, we decided to inject the cells through the walls of the decellularized esophagus with the help of a syringe. At 4 days, histology showed the presence of grouped and more rounded cells in the submucosal region of the esophageal matrix (Fig. 6A). But at days 7 and 14, the majority of cells were found near adventitia, but some cells were also observed near muscular, and submucosal area with evenly distributed and elongated cell types. At day 14, multilayered cells were observed around the adventitia of the esophagi (Fig. 6A-v, A-vi). Some MSCs were also differentiated to adipocyte-like cells near adventitia at day 14 time point (Supplementary Fig. S1). Prominent blue-colored cells were identified in the muscularis externa of the regenerated esophagus at day 14. Overall, the histological analysis showed signs of cell migration, survival, and growth in the decellularized esophageal tissue.

To investigate the total number of cells in the recellularized esophagus, we stained the tissue sections with DAPI and the average number of cells were counted in 10 randomly taken fields for each time point (Supplementary Fig. S2). As shown in the line graph, the recellularized tissues showed significant growth of the stem cells from days 4 to 14 (*p* < 0.05). The highest number of cells were found on day 14 followed by days 7 and 4.

The stem cell-seeded esophageal matrices were further investigated for stem cell differentiation and impact of structural proteins on the stem cell growth by immunofluorescence at day 14. As seen in Figure 6B, few cells were found to be differentiated into endothelial cells. In addition to this, smooth muscle cells were also identified in the recellularized esophagus (Fig. 6B-ii). Furthermore, expression of vimentin (a MSC marker) in the recellularized cells indicate that some cells still underwent differentiation. Strong expression of collagen IV, near recellularized cells, was identified but no expression of collagen I appeared near recellularized cells (Fig. 6B-iii).

**FIG. 7.**
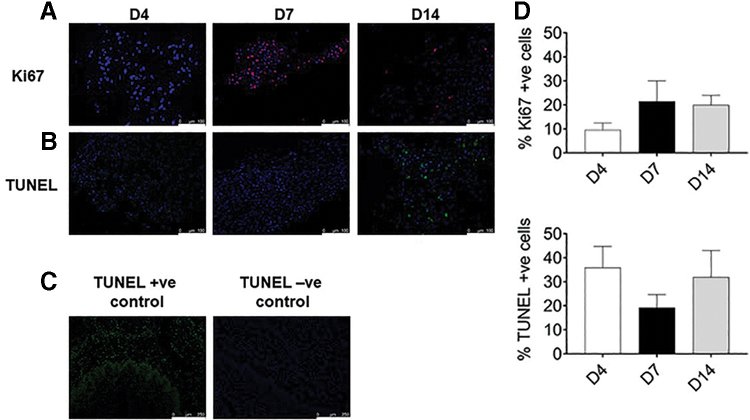
Immunofluorescence analysis and quantification of cell proliferation and apoptosis in the tissue-engineered humanized esophagus. **(A)** Immunofluorescence staining of the proliferation marker Ki67 (red) in recellularized esophagus tissues at days 4, 7, and 14. **(B)** Cell apoptosis was investigated by TUNEL assay in the recellularized esophagus tissues. Apoptotic cells were stained green and counterstained with blue-colored DAPI at days 4, 7, and 14. **(C)** TUNEL-positive control was induced by incubating tissue sections in DNase-I, where positive cells were seen in green color and in negative section no green-colored apoptotic positive cells. **(D)** Percentage of positive Ki67 and TUNEL cells in the tissues were counted by CellProfiler software. No significant difference was seen in Ki67 marker between days 4, 7, and 14. Nuclei (blue) was counterstained with DAPI. The results represent mean ± SEM, where *p* < 0.05 was considered as statistically significant using Kruskal–Wallis test followed by Dunn's multiple comparison *post hoc* test. Scale bars: 100 μm **(A, B)** and 250 μm **(C)**. TUNEL, terminal deoxynucleotidyl transferase dUTP nick end labeling.

We used Ki67 marker on the recellularized esophagi tissues to detect proliferation in the seeded stem cells. At day 4, 9.5 ± 2.9% of the cells were proliferating during recellularization (Fig. 7A, D), whereas at days 7 and 14, there was an increase in proliferating cells (21.5 ± 8.4% at day 7 and 20 ± 3.9% at day 14). However, the differences between days 4, 7, and 14 were not statistically significant.

To detect apoptosis during the recellularization procedure, we analyzed tissue samples which were taken at days 4, 7, and 14 with the TUNEL assay. At day 4, 35.9 ± 8.8% cells were apoptotic compared with 19.2 ± 5.4% at day 7; and 31.9 ± 11.1% at day 14 (Fig. 7B, D). There was no significant difference in the percentage of apoptotic cells in the recellularized tissue regardless of time point.

## Discussion

In the current research work, we successfully grew a TE esophagus, which showed that important ECM proteins were present in the decellularized esophageal scaffold that allows growth and differentiation of human amnion-derived stem cells in the scaffold. Only very weak activity against the Gal 1,3 Gal epitope remained.

Humans have natural antibodies against α-Gal epitopes of xenogeneic grafts. Transplantation of such xenografts into human or old world monkey would result in hyperacute rejection of the graft. In this study, we checked the expression of α-Gal epitopes in the decellularized tissue. Immunohistochemistry revealed that in comparison to normal esophagus, very weak expression of the α-Gal epitopes was seen in the SDC/DNase-I-based decellularized esophagus. It can be concluded that decellularization alone is probably sufficient to remove α-Gal epitopes from the cell surface but not from the ECM. Study has shown that treatment of an enzyme α-galactosidase on xenogeneic scaffolds could eliminate α-Gal epitopes from the animal tissue.^12^ According to a recent report, when decellularized porcine α-Gal knockout lungs and decellularized lung containing α-Gal epitope were recellularized with human lung cells, there was no difference found in terms of cell growth and proliferation in both the lung recellularization.^13^ Commercial glutaraldehyde-fixed heart valve transplantation exhibits anti-gal antibody immune reactions in humans and non-human primates. Alpha gal epitopes in these grafts were reported to be significantly higher compared with the gal knockout heart valves.^14^ However, glutaraldehyde-fixed heart valve grafts can remain viable for 8–10 years after implantation. We did not treat tissues with α-galactosidase or glutaraldehyde in this study.

The SDC/DNase-I (protocol 1), and Triton X/TnBP (protocol 2) have been successfully used for decellularization of tubular organs, such as blood vessels, trachea, and esophagus.^4–6^ The SDC solution is an ionic detergent, which solubilizes cell and nucleic membranes, whereas Triton X is a nonionic detergent which acts on lipid–lipid and lipid–protein bonds in cells in a relatively gentle manner. TnBP is an organic solvent which primarily acts on protein–protein interactions. Consequently, it is considered to be more disruptive toward tissue.^2^ In this study, we investigated the best possible combination of detergent/enzyme/physical methods to generate a successful decellularization protocol for porcine esophagus. Our SDC/DNase-I protocol was based on the previously established decellularization protocol for porcine esophagus.^6,8^ However, the previous SDC/DNase-I protocol was based on a small sample size, whereas our study utilized a larger sample size but with similar results.

Detergent treatments can damage tissue proteins and they are difficult to remove from the tissues after the decellularization process. This could eventually alter the recellularization process and could cause inflammatory reaction upon *in vivo* implantation. Hence, we also used the sonication/DNase-I protocol (protocol 3) to avoid the use of detergents for decellularization. Histological findings revealed that protocol 1 was superior in removing donor cells from the tissue using just three decellularization cycles, whereas protocols 2 and 3 were ineffective in removing donor cells despite employing 11 decellularization cycles. Our findings differ to those in a previous publication, where Triton X/TnBP/DNase-I was successfully used for decellularization of blood vessels.^5^ This organ-specific efficacy of Triton X/TnBP/DNase-I decellularization is probably due to the simple tubular form of a blood vessel compared with the thick and complex structure of esophagus. DNA quantification showed a significant reduction of donor DNA in protocol 1, whereas protocols 2- and 3-derived scaffolds had significantly higher donor DNA remaining. Based on histological and DNA quantification, the SDC/DNase-I-based protocol was the most successful in achieving complete decellularization of the esophagus.

To monitor presence of chemical residues in the decellularized esophagus, we also biochemically evaluated the presence of detergent. Using the current method,^15^ negligible detergent was found in decellularized tissues, which was not statistically different from that of normal untreated control, which contains lipids structurally identical to detergents. Our SDC/DNase-I protocol achieved 4% SDC which was effective in decellularization of esophagus in a shorter time. Although we found smaller amounts of SDC remnants in the tissue, we are unable to predict the potential reactions in *in vivo* implantation. In our decellularization protocol setup, we also tested a low concentration of SDC but it required more time to decellularize the tissue. The resultant tissue lacked the same mechanical strength as our protocol 1 decellularized esophagus. Due to the structural similarity of the lipid portion of tissue with detergent, the lipid content of tissues also reacted with sulfuric acid and produced coloration in normal untreated samples.

It is known that ECM proteins are well preserved in naturally obtained acellular biological scaffolds.^2^ Elastin and collagen are the structural proteins that play an important role in providing the mechanical properties of the tissue. Functionally, GAGs are pivotal for binding growth factors and cytokines, and to retain water molecules in the tissue. In this study, we quantified the amount of elastin, collagens (soluble and insoluble), and GAGs in the decellularized porcine esophagus. We found that protein content in decellularized tissue varied depending on the protein that was measured. When compared with normal tissue, we found that protocol 1 preserved elastin and insoluble collagen, whereas GAGs and soluble collagen were significantly reduced in the decellularized esophagus. Our finding supports previous reports on preservation of elastin and depletion of GAGs after an SDC-based decellularization protocol.^2,16^ Elastin is exclusively found in the ECM, whereas GAGs are found on the cell surface, in intracellular vesicles, as well as in the ECM. Decellularization is a process which removes cells from tissues or organs. Thus, removal of cellular components from the esophageal tissue resulted in a significant depletion of cell-bound GAGs compared with elastin. This finding was further supported by immunofluorescence analysis of ECM proteins in decellularized tissue, indicating preservation of elastin, collagen IV, fibronectin, and laminin (around the blood vessels only). Organization of collagen fibers in decellularized esophagus was similar to the native tissue as noted by MT and MP stains. Based on the ECM quantification and immunofluorescence analysis, the acellular scaffold displayed good mechanical and elasticity properties. One of the important properties of elastin is its capacity to stretch and return to its original state. This characteristic is of particular value to the esophagus which relies upon a constant peristaltic movement to be able to pass food and water into the stomach. We believe the recruitment of new stem cells during recellularization would be able to repair the damage caused to GAGs in decellularized tissue. Furthermore, our decellularization protocol successfully retained ECM proteins in the tissue, and could provide the ideal scaffold structure for organ generation through recellularization.

Surprisingly, we detected a donor cytoplasmic protein, that is, α-SMA in the decellularized tissue. This is in good agreement with our previous study and other studies on preservation of donor cytoplasmic proteins in the decellularized tissue.^13,16–18^ The preservation of α-SMA in different decellularized organs and species suggest that this effect is reproducible and neither organ nor species specific. The positive or negative effects of such cellular remnants in decellularized tissue during recellularization or in terms of host response during tissue implantation is unclear. However, increased α-SMA expression in fibroblast cells has been shown to increase contractibility of fibroblast cells.^19^ We speculate that residual α-SMA in decellularized esophagus provides cues to account for infiltrating MSCs during recellularization.

To investigate whether acellular esophageal scaffold could support stem cell growth and differentiation, we isolated human amnion-derived stem cells from human fetal membranes. In our study, expanded cells showed presence of epithelial and mesenchymal markers on the respective cell types as characterized by immunofluorescence analysis. The gradual loss of cobblestone-shaped epithelial cells and increased fibroblast-like cells in culture plates after passage 3, suggest a possible epithelial-to-mesenchymal transition (EMT) during epithelial cell expansion. This observation is in agreement with a previous report revealing increased vimentin (mesenchymal marker) expression on the 8th day of culturing amnion epithelial cells.^20^ A recent study, however, reported that amniotic membrane inhibits transforming growth factor-beta. This growth factor is a well-known EMT-inducing cytokine when cocultured with corneal and limbal fibroblasts.^21^ We hypothesize that EMT inhibitors should be used in epithelial cell cultures for future studies. These inhibitors may be able to prevent EMT during cell expansion.

Histological findings of the recellularized esophagus were evident at an early stage (day 4) of recellularization. Cells were attached and proliferated in the matrix, but most cells were clustered together and found near the adventitia and submucosal layer. At days 7 and 14, cells were observed on the outer adventitia and in a more diffused fashion. Very few epithelial cells were seen in the esophageal mucosa. Cell quantification showed significant growth of MSCs at day 14 and over days 4 to 7 in recellularized tissue. This finding highlights the benefits of a longer culture duration. These results, however, are in contrast to previous findings where perfusion recellularization effectively regenerated endothelial linings in the lumen of blood vessels.^5^ The parameters used in these latter studies, such as the scaffold preparation, stem cells source, and recellularization process, were not identical to ours. There are several possible reasons for the different results of whole-organ recellularization and re-epithelialization in our study. Positive or negative effects of perfusion–rotation bioreactor on the recellularization process is not fully understood, for example, reduced expression of GAGs in the acellular scaffold, which might be a plausible cause for the reduced cellular attachment of the whole organ and epithelial lining in the tissue as observed in our study.

Immunohistochemical analysis revealed differentiation of the seeded amnion stem cells into endothelial and smooth muscle cells on the recellularized esophagus. These results highlighted that ECM proteins and growth factors were retained in the acellular esophagus, and provided an effective criterion for differentiation of stem cells within the tissue. Moreover, the presence of vimentin in the recellularized tissue at day 14 suggest retention of MSC stem cell properties under long-term culture conditions. The differentiation and growth of MSCs in the collagen-rich ECM were earlier studied in mouse lungs by Daly et al.^18^ We observed the higher expression of collagen IV in regenerated tissue which was most likely associated with new proliferated cells, evidenced by immunofluorescence staining. Absence of collagen I marker in the recellularized esophagus culture at day 14 suggest nonfibrotic tissue remodeling.

In this study, we quantified proliferation and apoptosis of stem cells in recellularized esophagus. At day 4, more apoptotic and fewer proliferative cells suggest an early cell death and inadequate proliferation in the scaffold. Compared with day 4, we found ∼2.3-fold increase in cell proliferation at days 7 and 14. This observation was surprising given that cells had proliferative capacity even after 2 weeks. The observation further confirms the nontoxic nature of the acellular scaffold. We speculate that the higher oxidative stress in recellularized tissue at day 14 could have caused higher apoptosis in the tissue. We did not see differentiation of MSCs into striated muscle cells in recellularized tissue. Previous reports on regeneration of epithelial and muscular regions of esophagus in small and large animal models show the possibility of *in vivo* regeneration of partially recellularized natural or artificial scaffolds.^22–24^ This tissue-specific differentiation of cell types in recellularized tissue suggest that engineered esophagus would be suitable for future *in vivo* applications.

There are few limitations associated with this study. It is important to mention that treatment of α-galactosidase or glutaraldehyde to acellular esophagus in this study could have removed α-Gal from the tissue. Chemical methods of decellularization usually cause damage to the ECM and prevent further cellular repopulation. Reduced cellular repopulation, modified cellular architecture, and failure to regenerate complete mucosal lining of the esophagus as observed in our study suggest further refinement and modification of chemical protocols in future studies.

During the current study, we have successfully prepared and characterized de- and recellularized esophagus suggesting its future potential use in the clinic. Altogether, our results are encouraging and provide a better understanding toward achieving complete recellularization and a functional esophagus suitable for transplantation.

## Supplementary Material

Supplemental data

## Abbreviations Used

BSA: bovine serum albumin
CO_2_: carbon dioxide
DAPI: 4’,6-diamidino-2-phenylindole
DMEM: Dulbecco's modified Eagle's medium
DW: distilled water
ECM: extracellular matrix
EMT: epithelial-to-mesenchymal transition
GAGs: glycosaminoglycans
H&E: Hematoxylin and Eosin
MP: Movat's pentachrome
MSCs: mesenchymal stem cells
MT: Masson's trichrome
NS: not significant
PBS: phosphate-buffered saline
RT: room temperature
SDC: sodium deoxycholate
SEM: standard error of mean
SMA: smooth muscle actin
TE: tissue engineered
TnBP: Tri-n-Butyl phosphate
TUNEL: terminal deoxynucleotidyl transferase dUTP nick end labeling
