# Supplementary material for: In Vitro Regeneration of Decellularized Pig Esophagus Using Human Amniotic Stem Cells
Source: Biores Open Access. 2020 Feb 21;9(1):22–36. doi: 10.1089/biores.2019.0054 (PMC7047253; doi:10.1089/biores.2019.0054)
Supplement: Supplemental data [file Supp_Data.pdf]

Supplementary Data

DNA Quantification

The double-stranded DNA (dsDNA) isolation was performed according to the manufacturer's (DNeasy Blood and Tissue Kit; Qiagen, Switzerland) instructions. Briefly, lyophilized tissue samples ($\sim$10–15 mg, protocol 1: $n=9$, protocols 2 and 3: $n=5$) were incubated overnight in proteinase K at 56°C. Buffers provided with kit were added to the samples and centrifuged in a DNeasy mini spin column to selectively bind the DNA to DNeasy membrane (silica membrane based). Contaminants and enzyme inhibitors were removed by two efficient washing steps. The purified DNA was further treated with fluorescent nucleic acid stain and quantified with the Quant-iT PicoGreen dsDNA Kit (Molecular Probes, Eugene, OR) according to the kit manual and concentration of the dsDNA was measured on a plate reader (Varioskan Lux-Thermo Scientific, ScanIT 4.1 software).

Isolation, Expansion, and Characterization of Cells for Recellularization

Epithelial cells

In brief, immediately after collecting term placental tissues, attached amniotic membranes ($n=9$) were transported the 10 km distance from the Östra Hospital, Gothenburg, Sweden to the laboratory in an ice-cold phosphate-buffered saline (PBS), containing 1% antibiotics and antifungal. After receiving the tissue sample, all further procedures were performed in a laminar air flow hood. The amnion (AM) tissue was separated from the chorionic part with blunt dissection, and cut $\sim$2 cm from the placental disc. AM was then washed extensively with PBS containing 1% antibiotics and antifungal and then chopped into 2×2 cm pieces. Each chopped piece of AM was incubated in 20 mL of Dulbecco's modified Eagle's medium (DMEM; Lonza, Verviers, Belgium) medium supplemented with 0.1% trypsin ethylenediaminetetraacetic acid (EDTA) and 1% antibiotics and antifungal, and kept for digestion at 37°C for 20 min with gentle agitation. Enzyme activity was neutralized by adding 50 mL DMEM with 10% human AB serum (Sigma-Aldrich, St. Louis, MO) and the suspension was passed through a 100 μm filter. Cells were obtained by centrifugation of the filtrate for 1500 rpm for 10 min. This procedure was repeated twice for each 2×2 cm piece of AM and cells were pooled together. Epithelial cells were then counted and cultured in 0.2% gelatin-coated culture plates with DMEM and F12 media in 1:1 proportion, supplemented with 10% heat inactivated human AB serum, 1% L-glutamine, 1% penicillin/streptomycin, the HCM Single Quote Kit (Lonza) containing ascorbic

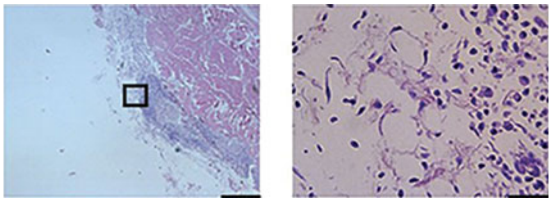

SUPPLEMENTARY FIG. S1. Adipocyte-like cells differentiated in tissue-engineered esophagus. Hematoxylin and Eosin-stained recellularized esophagus tissue with stem cell growth at 2 weeks (left panel). The black box in the left panel is the area in the right panel in 400× magnification. Adipocyte-like cells in the 400× magnified image (right panel). Scale bars: 750 μm (left panel) and 50 μm (right panel).

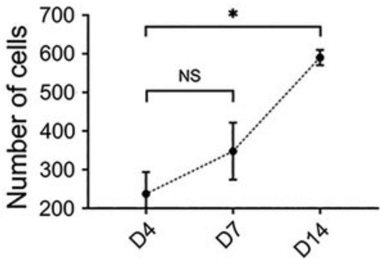

SUPPLEMENTARY FIG. S2. Quantification of seeded stem cells in the recellularized esophagus. Recellularized esophageal tissue sections at days 4, 7, and 14 were stained with DAPI. The DAPI-positive cells per field were counted in 10 randomly taken images ($n=5$). Error bars represents standard error of mean. p-Value <0.05 was considered as statistically significant using Kruskal–Wallis test followed by Dunn's multiple comparison post hoc test. *Represents $p=0.0267$. DAPI, 4′,6-diamidino-2-phenylindole; NS, not significant.

Supplementary Table S1. List of Antibodies, Working Dilution, and Manufacturer's Name

Sr. No.	*Antibody name*	*Host species*	*Working dilution*	*Manufacturer and product code*
1.	Elastin	Mouse	1:100	Abcam, Cambridge, UK; ab9519
2.	Laminin	Rabbit	1:200	Abcam; ab11575
3.	Fibronectin	Rabbit	1:200	Abcam; ab23751
4.	Collagen I	Mouse	1:250	Abcam; ab23446
5.	Collagen IV	Rabbit	1:250	Abcam; ab6586
6.	α-SMA	Rabbit	1:100	Abcam; ab5694
7.	Tropomyosin	Mouse	1:50	Sigma-Aldrich, St. Louis, MO; T9283
8.	Gal-alpha 1–3 gal	Mouse	1:5	Salem, MA; UG1025-10
9.	Vimentin	Mouse	1:500	Abcam; ab20346
10.	EpCAM	Mouse	1:200	Abcam; ab46714
11.	Ki67	Rabbit	1:200	Abcam; ab15580
12.	CD31	Rabbit	1:100	Abcam; ab28364
13.	Alexa Fluor 488 against mouse	Goat	1:400	Abcam; ab150117
14.	Alexa Fluor 488 against rabbit	Goat	1:400	Abcam; ab150088

EpCAM, epithelial cell adhesion molecule; SMA, smooth muscle actin.

acid, bovine serum albumin/fatty acid free, hydrocortisone, transferrin, insulin, recombinant human epidermal growth factor, and gentamicin sulfate. After 24 h, the medium was discarded and the culture plate was washed twice with PBS to remove floating cells. Epithelial cells were expanded until passage 3 and were stained for antiepithelial cell adhesion molecule marker to confirm epithelial phenotype before recellularization.

Mesenchymal stem cell

The AM was placed in a container with 20 mL of DMEM (Lonza) medium supplemented with 0.1% trypsin EDTA and 1% antibiotics and antifungal. The mixture was left at 37°C for 20 min and gently agitated to form a digest. The digest solution was then discarded to remove epithelial cells from AM pieces. The AM pieces were subjected to further digestion in 50 mL DMEM supplemented with 0.75% collagenase type II (Sigma-Aldrich), 1% antibiotics, and antifungal at 37°C for 30 min with gentle agitation. The digest was filtered through a 100 μm mesh strainer, and the filtrate neutralized with 50 mL DMEM with 10% human AB serum. Mesenchymal stem cell (MSC's) were obtained by centrifugation of the filtrate at 1500 rpm for 10 min. At the end of the process, cell viability was checked on a cell counter and cells were plated and grown in complete MesenPRO media (Life Technologies, NY) until passage 5. MSC's were subsequently stained for dual-color immunofluorescence histology using antibodies to vimentin (Abcam, Cambridge, UK) and counterstained with 4',6-diamidino-2-phenylindole in a chambered slide to confirm cell phenotype before recellularization.

Estimation of detergent residues in acellular esophagus

The method published by Urbani and Warne[S1] was used with slight modification for the estimation of detergent in decellualrized esophagus. Briefly, normal and decellularized tissue lysates corresponding to 50–100 μg of protein from normal and decellularized esophagus were treated with 800 μL of concentrated sulfuric acid, vortexed, allowed to cool for a period of 5 min, and finally centrifuged at 2000 rpm. The absorbance of the supernatant generated at 389 nm was plotted against sodium deoxycholate standard at different concentrations.

Supplementary Reference

S1. Urbani A, Warne T. A colorimetric determination for glycosidic and bile salt-based detergents: applications in membrane protein research. Anal Biochem. 2005;336:117.
